# Role of international network on surveillance and response system leading to malaria elimination: China’s engagement in global health

**DOI:** 10.1186/s40249-022-00991-z

**Published:** 2022-06-04

**Authors:** Shenning Lu, Lulu Huang, Lei Duan, Qiuli Xu, Xuejiao Ma, Wei Ding, Duoquan Wang, Shan Lv, Ning Xiao

**Affiliations:** 1 Chinese Center for Disease Control and Prevention (Chinese Center for Tropical Diseases Research), NHC Key Laboratory of Parasite and Vector Biology, WHO Collaborating Centre for Tropical Diseases, National Center for International Research On Tropical Diseases, National Institute of Parasitic Diseases, Shanghai, 200025 China; 2 Pudong New Area Center for Disease Control and Prevention and Pudong Institute of Preventive Medicine, Fudan University, Pudong New Area, Shanghai, 200136 China; 3 School of Global Health, Chinese Center for Tropical Diseases Research, Shanghai Jiao Tong University School of Medicine, Shanghai, 200025 China; 4 State Key Laboratory of Genetic Engineering, Ministry of Education Key Laboratory for Biodiversity Science and Ecological Engineering, Ministry of Education Key Laboratory of Contemporary Anthropology, Department of Infectious Diseases, School of Life Science, Huashan Hospital, Fudan University, Shanghai, 200433 China

**Keywords:** Surveillance and response system, Malaria, Elimination, Network

## Abstract

China has accumulated multiple practices and experiences in building and enhancing malaria surveillance and response system. As China’s engagement into global health has gathered stronger momentum than ever, China together with the Swiss Tropical and Public Health Institute and WHO has organised five sessions of the International Forum on Surveillance-Response System Leading to Tropical Diseases Elimination during 2012–2020, in which malaria elimination has always been one of the hottest topics. In this study, the roles of international network on the surveillance and response system were explored to achieve a global malaria-free goal. China’s approach to malaria elimination has demonstrated significance of global collaboration on taking joint prevention and control, and building a worldwide institutional-based network.

## Background

Malaria, an ancient and life-threatening mosquito-borne disease, has continued to threat people’s health and livelihood. Momentum towards renewed response to malaria in the globe has been building since the turning of the new century, after the first Global Malaria Eradication Programme failed in the mid-twentieth century [1]. Reignited political commitment, stepped-up investment in research and innovation and creation of new financing mechanisms enable unprecedented progress, with global malaria death steadily declining from 896,000 in 2000 to 627,000 in 2020 [2]. Malaria eradication is back on the table, as global stakeholders such as the World Health Organization (WHO), the Rolling Back Malaria Partnership to End Malaria [3], the Global Fund to Fight AIDS, Tuberculosis and Malaria, the US President’s Malaria Initiative, the Bill and Melinda Gates Foundation and many others reckoned that malaria elimination is within reach with concerted efforts [4]. A set of global strategies and a potential timeline were put forward [1]. Notably, the Global Technical Strategy for Malaria 2016–2030 (GTS) and the Action and Investment to defeat Malaria 2016–2030 were adopted in 2015, setting the ambitious new target of reducing the global malaria burden by 90% by 2030 [3, 5].

Despite major achievements in the past two decades, recent 4 years have witnessed the world stuck in a plateau. According to the World Malaria Report 2021, an estimated 241 million malaria cases occurred leading to 627,000 deaths in 2020, most critically affecting sub-Saharan Africa [2]. Additionally, the future of malaria seems bleak under the COVID-19 pandemic, which puts strains on the malaria interventions worldwide. The gains made in saving lives from over the past 20 years could be lost, unless all of the WHO guidelines against both the COVID-19 pandemic and malaria are followed [6].

Surveillance and response have been recognized as crucial steps in effective control and elimination of tropical diseases, malaria included [7]. Malaria surveillance has transformed into and become a key intervention under the Pillar 3—transform malaria surveillance into a core intervention according to GTS [5]. By providing timely, specific data and information at a national level or certain geographical areas, the surveillance system is pivotal to empower a more focused malaria response [8].

China has made tremendous progress towards malaria elimination—China has been certified as malaria-free country by WHO on June 30, 2021. With a robust and improved malaria surveillance and response system (SRS) network both domestically and internationally, China is able to not only secure and maintain a malaria-free future within its border but also has huge potential to contribute to global malaria elimination efforts [8, 9]. This is a review of the international network on SRS led by China and its implications for malaria elimination.

### The role of the SRS in malaria elimination

Surveillance of infectious disease is recognized as the cornerstone of public health decision-making and practice. Weak surveillance system stands as one of challenges for malaria elimination. Malaria surveillance has been defined as to collect data in a systematic way, consolidate information and deliver it quickly to guide the decision making towards malaria prevention and control actions [10]. In recent years, even though the surveillance systems in most high-burden countries have been considerably improved, they still fail to capture essential malaria data in a complete, accurate and timely manner, thus making it difficult to optimize response plan, evaluate the diseases trends, inequalities, and gaps in interventions, and respond to outbreaks [11]. A robust SRS can effectively identify specific high-risk populations, determine their size, and track them over time with adequate representativeness to accurately assess the rates of infection, and the use of preventive measures. Surveillance may function most intensively as an intervention while the programme is the closest to elimination and effective surveillance is required at all points on the path to elimination.

### China’s surveillance-response towards malaria-free future

With 30 million cases of the disease annually in the 1940s and no indigenous malaria cases since 2017, China became the 40th country and the most populous one to be certified malaria-free by WHO [12, 13]. Practices and experiences in building and enhancing the malaria SRS in China’s 70-year effort could be shared with the malaria epidemic countries which face gaps in surveillance coverage, health information architecture, data capture, and etc. [14].

In China, the SRS has been well integrated within its public health system [15]. From the beginning of the national malaria control programme in the 1950s, a nationwide malaria surveillance system was gradually established and later reinforced in 2005 when 62 sentinel sites were set up [16]. As China entered malaria elimination phase in 2010, the focus of surveillance and response has shifted to individual cases and foci. In 2012 the national surveillance systems was initiated which comprises routine surveillance and sentinel surveillance [17]. For the routine surveillance, a national network of case reporting and case management is formed from the county level, municipal level, provincial level to national level, while entomological surveillance, antimalarial drug resistance surveillance, surveillance on population at risk are carried out as a sentinel surveillance. The “1-3-7” norm (case notification within 1 day, case investigation within 3 days and focus disposal within 7 days) was adopted to rapidly detect and identify all malaria infections and ensure an appropriate treatment before any secondary infected cases or local transmission may occur. The national roll-out of the “1-3-7” norm presented with good performance, with all cases reported within 24 h after diagnosis, 97.9% (2619/2674) of them investigated on epidemiology within 3 days, and 2326 foci identified, investigated and responded to within 7 days in 2019 [18]. Recognized by WHO, the “1-3-7” norm has been recommended as a surveillance and response strategy within the Greater Mekong Subregion, including Cambodia and Thailand, as well as that been modified as 1,7-malaria Reactive Community-based Testing and Response approach in the context of Tanzania [19, 20].

The surveillance and response network in China plays a crucial role in identifying public health problems, ascertaining the distribution and epidemic dynamics of malaria nationwide, detecting outbreaks and epidemic anomalies, evaluating the effects of on-site interventions and identifying risk factors as well as at-risk populations and regions.

### A case study of the International Forum on the SRS

China’s engagement into global health has gathered strong momentum. Following the establishment of public health network for cooperative opportunities launched in 2017 during the Belt and Road High-Level Meeting, the Institutional-based Network of China-Africa Cooperation on Malaria Elimination (INCAM) was founded at the High-Level Meeting on China-Africa Health Cooperation in the following year. China has begun to collaborate with the international community to call for a wider network on the SRS, given its experience in applying innovative genetics-based approaches and tools which could offer game-changing results when applied to other settings with ongoing transmission, and even on the path to malaria elimination [21]. As the WHO Collaborating Centre for Tropical Diseases, the National Institute of Parasitic Diseases (NIPD) at Chinese Center for Disease Control and Prevention (China CDC) has been playing an important role in organizing and coordinating the activities of both domestic and international network on the SRS [22].

The International Forum on Surveillance-Response System Leading to Tropical Diseases Elimination (ISRS) is an exemplary event in advocating the international network on malaria SRS. Bringing together scientists in research, public health and health policy, this forum is jointly supported by NIPD at China CDC, the Swiss Tropical and Public Health Institute and WHO. ISRS aims to share the knowledge and experiences pertaining to the control, prevention and elimination of major tropical diseases and to discuss novel approaches towards the establishment of an integrated surveillance-response platform and network, in which malaria is listed high on the agenda. In-depth discussion was held on current challenges, opportunities, and measures to foster reliable effective SRS as a strategic key for endemic countries moving towards the elimination. After the first ISRS in 2012, the participants suggested that the forum should be convened every 2 years, to keep abreast of the latest research priorities and discuss challenges in implementing local, national, or regional SRS.

As of 2020, a total of five ISRS have been organized, which have gained increasing attention and impacts within the international malaria and NTDs community. The number of stakeholders who participated in the ISRS is estimated to grew a tenfold from around 100 from 10 countries in 2012 to 1000 from 40 countries in 2020. The surge of the participants in the latest forum was mainly contributed to the live broadcast as the consequence of COVID-19 pandemic, which allowed the ISRS reach a broader range of the participants. The agenda for discussion on the malaria SRS in each ISRS closely followed the mandates and echoed the initiatives of the important international cooperative channels such as Brazil, the Russian Federation, India, China and South Africa (BRICS) Summit and the Forum on China-Africa Cooperation, in addition to the regular updates (Table 1). The items on agenda also took consideration of the current global situation such as the COVID-19 pandemic and promptly initiated conversation on how to address the double challenges human faces. Furthermore, the ISRS provided an institution-oriented platform for exchanging ideas and generating new collaborations and partnerships on malaria, through which multiple memoranda of understanding (MoU) have been signed between NIPD at China CDC and its counterparts. Bi-lateral and multi-lateral international cooperation were also consolidated and expanded, including pilot cooperation projects on malaria control in Tanzania and Papua New Guinea. Scientific outcomes of the ISRS included publications on the topics of the SRS at the national, regional, and international scale as one of key indicators to promote the control and elimination of tropical diseases. What’s more, the Infectious Diseases of Poverty, a unique journal that fills that communication gap between developing and developed countries and encourages research breakthroughs about surveillance and response was first presented through the ISRS.

**Table 1:** An overview of five ISRS

Time	Presentations related to malaria SRS	Outputs
1st ISRS (2012)	-How to establish more effective and novel SRS in tailored to the various disease endemic areas	-1 summary report -4 research priorities -Inauguration of IDP - 1 MoU signed with LSHTM
2nd ISRS (2014)	-Significance of surveillance-response system in BRICS malaria elimination programmes -Harnessing China-Africa Cooperation Initiatives in Malaria Control and Elimination	-1 summary report -4 MoUs signed with TBRI, IHI, DGHI, Fudan
3rd ISRS (2016)	Exploring the bilateral and multilateral cooperation aiming at malaria control and elimination in Africa	-1 report on the China-UK-Tanzania pilot cooperation project for malaria control –1 report on China-Australia-Papua New Guinea pilot trilateral cooperation project for malaria control
4th ISRS (2018)	-Innovative technology to improve the SRS through platforms supported by essential databases -Application in national control and elimination programmes on malaria -New South-South cooperation on pilot studies in application of the SRS	-4 MoUs signed with Cameroon, Ethiopia, Sierra Leone, Zambia -Establishment of INCAM
5th ISRS (2020)	-Review the progress and challenges due to COVID-19 pandemic -Progress of malaria elimination in Greater Mekong Region -China-Myanmar collaboration for malaria elimination in the border areas -Enhancing INCAM communication mechanism -Setting up INCAM academic committee	-INCAM communication mechanism

*SRS* surveillance and response system, *ISRS* the International Forum on Surveillance-Response System Leading to Tropical, *BRICS* Brazil, the Russian Federation, India, China and South Africa Summit, *MoU* memorandum of understanding, *TBRI* Theodor Bilharz Research Institute, *IHI* Ifakara Health Institute, *IDP* infectious diseases of poverty, *LSHTM* London School of Hygiene and Tropical Medicine, *DGHI* Duke Global Health Institute, *INCAM* the Institutional-based Network of China-Africa Cooperation on Malaria Elimination

## Discussion

The ISRS serves as a window to recap the current status of China’s malaria SRS, disseminate the latest scientific and relevant outputs in this field, as well as identify the urgent challenges in the face of the world. Chinese representatives from multi-sectors including policy makers from the National Health Commission, experts from national and provincial institute of parasitic diseases and other related scientific research institutes, frontline health workers, universities, enterprises, etc. have been brought together with other partners of the world. On the one hand, whether the national malaria programmes are progressing as planned or whether adjustments in scale are required have been assessed. Moreover, innovative approaches and cutting-edge tools have been introduced and presented through this platform, which allowed professionals in building and running the national malaria SRS on the same page and make informed decisions. Meanwhile under the ISRS, the obstacles to better implementation of the China’s malaria SRS were pointed out and possible solutions were discussed and found in a timely manner. In this sense, any efforts from the top to the bottom in China were synergized to safeguard the hard-won success of malaria elimination by focusing the key pillar of the SRS.

The ISRS provides a resource-sharing platform for inspiring an effective cooperation on the SRS between Asia Pacific and Africa, so as to help partner countries move forward to malaria elimination. Regional collaboration is one of the most important components to facilitate cross-border communication and coordination efforts, to address the transmission foci that cross international boundaries and to resolve common bottlenecks. China’s experience and resources in malaria elimination have been shared by such an exchange mechanism, such as the “1-3-7” norm. Being spread and recognized through the ISRS platform, the “1-3-7” norm has since been adapted or tailored to the local contexts in several country settings in Southeast Asia and Africa, including Myanmar, Cambodia, Tanzania among many others, with promising results yielded. Besides the positive contribution to malaria elimination efforts in partner countries, the ISRS to some extent benefits China in preventing re-establishment of infection diseases. Even after the malaria has been eliminated in China, continued importation of malaria cases may jeopardize China’s achievement, posing challenges to the SRS. Therefore, enhanced collaboration and coordination efforts with neighboring countries and Africa are necessary to keep vigilant against potential outbreaks caused by imported cases [23].

A salient role of the ISRS leading to malaria elimination is to innovate an institution-based network of surveillance and response, building up valuable relationships with potential new members, partners of the forum, and external stakeholders active in the field of malaria. The goals of malaria control and elimination may never be achieved without a strong involvement of those scientists and support of their institutions. The number of the MoUs between NIPD and institutions from home and abroad throughout the past 8 years has been on the rise, and meanwhile the geographical area and collaboration content have been expanded in an astonishing way as well. The ISRS has demonstrated successful in fostering networking between researchers of different disciplines and countries. Key stakeholders such as funding agencies are also fully engaged in the network, where new collaboration and partnership are encouraged. It is the multilateral communication and exchange mechanism on malaria SRS characterized by specialized research institutions that have attracted more actors across the continents and calls for concerted efforts to the cause of malaria eradication.

Compared with the Asia Pacific Leaders Malaria Alliance, Asia Pacific Malaria Elimination Network, two existing regional malaria platforms China has joined in, ISRS is the first international network proposed and initiated by China, with a key focus on developing and coordinating surveillance and response activities. The development of ISRS faces challenges such as setting up a clear organizational structure, building a smooth working mechanism and obtaining long-term financial support.

## Conclusions

Even though officially awarded malaria-free certification by WHO, China is still at the high stake of imported malaria cases since it borders with the countries with high malaria burden and it has a large migrant worker community which often work in Africa. China could not only rely on a strong domestic malaria SRS to prevent the reestablishment of the disease, but also need a powerful international network. The ISRS led by the institutions from China, Switzerland, and WHO represents a new cooperation mechanism making impacts on the Southeast Asia and Africa, to share the best practices and experiences of the SRS from China, partner countries and the international community, facilitate regional collaboration on joint prevention and control, spawn an institution-based network of surveillance and response. Continued technical and financial support is urgently required to sustain the international network on the SRS leading to global malaria elimination including strengthening a well-coordinated communication mechanism, providing an innovation and research exchange platform, and giving full play of interventions and policy recommendations.

## Data Availability

The datasets during and/or analysed during the current study available from the corresponding author on reasonable request.

## Abbreviations

SRS: Surveillance and response system
WHO: World Health Organization
GTS: Global technical strategy
INCAM: Institutional-based Network of China-Africa Cooperation on Malaria Elimination
CDC: Chinese Center for Disease Control and Prevention
ISRS: International Forum on Surveillance-Response System Leading to Tropical Diseases Elimination
NIPD: National Institute of Parasitic Diseases
BRICS: Brazil, the Russian Federation, India, China and South Africa Summit
